# Enhanced Stability of Extracted Sour Cherry Anthocyanins Copigmented With Tannic Acid and Encapsulated by Spray Drying

**DOI:** 10.1002/fsn3.70365

**Published:** 2025-05-30

**Authors:** Shirin Salati, Niloofar Moshfegh, Farzaneh Vaseghi‐Baba, Mehrdad Niakousari, Seyed Mohammad Mazloomi, Seyed Mohammad Hashem Hosseini, Azam Abbasi

**Affiliations:** ^1^ Department of Food Hygiene and Quality Control, School of Nutrition and Food Sciences, Nutrition Research Center Shiraz University of Medical Sciences Shiraz Iran; ^2^ Department of Food Science and Technology, School of Agriculture Shiraz University Shiraz Iran

**Keywords:** anthocyanin, copigmentation, encapsulation, stability

## Abstract

Anthocyanins are valuable natural colorants but are inherently unstable when exposed to external factors. This study aimed to enhance the stability of sour cherry anthocyanins through copigmentation with tannic acid (TA) at a ratio of 1:0.25 (anthocyanin:TA), followed by encapsulation using maltodextrin (MD) or a combination of MD and Arabic gum (AG) in a 90:10 (w/w) ratio via spray drying. The resulting powders exhibited high encapsulation efficiency (97.27%–98.92%), low water activity, and excellent solubility (> 99.98%). Among the treatments, the copigmented anthocyanin powder encapsulated with the MD‐AG mixture showed significantly higher a* and chroma values, indicating a more intense red coloration. After 28 days of storage under three different conditions, the copigmented samples retained significantly more anthocyanin compared to noncopigmented samples. In model beverages, color stability remained consistent over 49 days of storage for both the encapsulated powders and the controls (anthocyanin extracts with or without TA). In conclusion, copigmentation with TA combined with encapsulation using MD‐AG wall materials presents an effective strategy for enhancing the stability and color retention of sour cherry anthocyanins.

## Graphical abstract

Anthocyanins are unstable against external factor. Sour cherry anthocyanins copigmented with tannic acid and encapsulated by maltodextrin and Arabic gum as wall material. Copigmentation and encapsulation could stabilize red color and preserving anthocyanin.

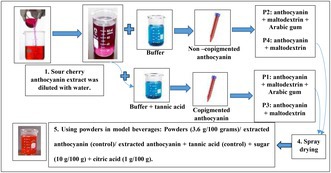

## 1 Introduction

The visual characteristics of food, particularly color, play a crucial role in shaping consumer expectations and satisfaction (Silva et al. 2022). Food colors are additives incorporated into foods and beverages for various purposes, such as enhancing appearance, standardizing color, and indicating flavor or quality (Sezgin and Ayyıldız 2017). Although synthetic colorants offer advantages, such as ease of use and a wide range of hues, they are often associated with potential health risks, including carcinogenicity, mutagenicity, skin irritation, allergic reactions, and respiratory problems (Amchova et al. 2024; Oplatowska‐Stachowiak and Elliott 2017). Consequently, there has been a growing demand for natural colorants in recent years (Neves et al. 2021).

Anthocyanins, a class of flavonoids, are widely used as natural food pigments due to their vibrant hues and recognized health benefits (Khoo et al. 2017; Salehi et al. 2020). However, their application is limited by poor stability. Anthocyanins are highly sensitive to various environmental and processing factors such as pH, light, temperature, sulfites, ascorbic acid, oxygen, and enzymatic activity (Enaru et al. 2021). This instability presents a significant challenge for food manufacturers working with complex food systems that incorporate both natural and synthetic matrices (Chen et al. 2023).

To address this limitation, two major strategies, copigmentation and encapsulation, are commonly employed to enhance anthocyanin stability (Darijani et al. 2025; Gençdağ et al. 2022; Xue et al. 2019). Copigmentation refers to the self‐association or interaction of anthocyanins with other compounds to improve antioxidant activity, enhance anthocyanin stability, protect flavylium cations from nucleophilic water attack, and intensify color expression (Li, Bao, et al. 2024; Li, Feng, et al. 2024; Tan et al. 2021). The effectiveness of copigmentation is influenced by several factors, including the type and concentration of anthocyanins and copigments, their molecular structures, pH, temperature, and solvent composition (Dai et al. 2022; Trouillas et al. 2016). Common copigments include phenolic acids, amino acids, alkaloids, and organic acids. In aqueous, mildly acidic conditions, the addition of a colorless copigment to anthocyanins typically results in a significant intensification of color, characterized by a hyperchromic shift (increased absorbance) and a bathochromic shift (movement of maximum absorbance to longer wavelengths) (Lv et al. 2022). Several studies have confirmed that copigmentation with phenolic acids enhances both the vibrancy and stability of anthocyanins (Eiro and Heinonen 2002; Fan et al. 2019; Zhu et al. 2020). Tannic acid (TA), a hydrolyzable phenolic compound, has demonstrated particularly strong hyperchromic and bathochromic effects when used as a copigment with anthocyanins (Molaeafard et al. 2021).

Encapsulation, especially via spray drying, is another effective method for stabilizing anthocyanins, enabling their incorporation into aqueous and other sensitive systems (Kaderides et al. 2024). Common wall materials used for encapsulation include maltodextrin (MD), Arabic gum (AG), and emulsifying starches (Darijani et al. 2025; Mahdavi et al. 2016). Since no single coating material can fulfill all functional requirements for optimal encapsulation, combinations of wall materials are often employed to improve encapsulation efficiency, solubility, and stability (Burin et al. 2011; Turchiuli et al. 2005).

AG is water‐soluble and primarily composed of carbohydrates (Chung et al. 2016). Xue et al. (2019) demonstrated that encapsulating copigmented anthocyanins from red‐fleshed apples using a combination of AG and MD enhanced color intensity, improved encapsulation efficiency, and increased anthocyanin stability. Similarly, Deng et al. (2023) evaluated the stability of purple corn anthocyanins encapsulated with MD, both alone and in combination with AG and whey protein isolate. Their findings revealed that the MD–AG combination provided superior protection against light degradation and significantly extended the half‐life of the encapsulated anthocyanins.

Sour cherries are rich in both noncolored polyphenolic compounds such as flavonols (primarily neochlorogenic acid) and colored phenolics, mainly anthocyanins (Kaur et al. 2023; Molaeafard et al. 2021). The major anthocyanins consistently reported in sour cherry include cyanidin‐3‐glucosyl‐rutinoside, cyanidin‐3‐rutinoside, cyanidin‐3‐sophoroside, and cyanidin‐3‐glucoside (Bonerz et al. 2007; Cairone et al. 2023; Toydemir et al. 2013). However, the color quality of these compounds is highly sensitive to heat, oxygen, light, and other processing or storage conditions (Ribárszki et al. 2022; Szalóki‐Dorkó et al. 2015).

Given their potential as natural substitutes for synthetic colorants and their inherent instability, the present study aimed to extract anthocyanins from sour cherries and improve their stability through copigmentation with TA, followed by encapsulation using MD alone or a mixture of MD and AG via spray drying. The study evaluated the effects of copigmentation and encapsulation on key physicochemical properties and the storage stability of the resulting powders, including their performance in a model beverage system.

## 2 Materials and Methods

### 2.1 Materials

Sour cherries, AG, and sugar were purchased from a local market in Shiraz, Iran. MD (dextrose equivalent = 11) was obtained from Mixadd (Iran), and TA was sourced from Molekula (UK). All other chemicals used in the study were of analytical grade.

### 2.2 Anthocyanin Extraction From Sour Cherries

Fully ripened sour cherries ( *Prunus cerasus* L.) were pitted and stored at −18°C until extraction. The extraction solvent was prepared by mixing 96% ethanol with 0.1% hydrochloric acid (HCl). Thawed cherries were crushed and combined with the extraction solvent at a 1:1 (v/v) ratio. The mixture was allowed to stand for 3 h and then centrifuged at 8000 × *g* for 30 min (Sigma 3K30, Germany). The resulting supernatant was filtered through Whatman No. 1 filter paper using a Büchner funnel under vacuum. The filtered extract was concentrated at 45°C under reduced pressure using a rotary evaporator (Hei‐VAP Core, Heidolph, Germany) and stored at −18°C in Falcon tubes for further analysis (Moshfegh et al. 2025).

### 2.3 Preparing Copigment Solution and Calculation of Copigmentation Effect

Based on the findings of Moshfegh et al. (2025), a molar ratio of 1:0.25 (anthocyanin:TA) was used for the copigmentation process. Briefly, the extracted sour cherry anthocyanin was diluted with distilled water at a ratio of 1:10 (v/v). Separately, TA solution (0.015 M) was prepared in a phosphoric acid–sodium acetate buffer (pH 3.5). This TA solution was then mixed with the diluted anthocyanin extract at a 1:1 (v/v) ratio, resulting in a final anthocyanin‐to‐TA molar ratio of 1:0.25. Both the copigmented solution and the control (anthocyanin solution without TA) were kept in the dark for 1 h prior to further analysis.

To evaluate the copigmentation efficiency, two key parameters were assessed:

Hyperchromic effect (%) $\left(∆A=\frac{A-A_{0}}{A_{0}}\times 100\right)$, which indicates the increase of absorption of copigmented sample (*A*) in comparison with the sample without copigmentation (*A*_0_), and bathochromic shift $\left(∆\lambda_{\max}=\lambda -\lambda_{0}\right)$, which indicates the difference between the maximum absorption wavelength of the copigmented sample (*λ*) and the noncopigmented sample $\left(\lambda_{0}\right)$.

### 2.4 Spray Drying

An initial mixture of anthocyanin:TA at a molar ratio of 1:0.25 with Brix 3 was prepared. To achieve a Brix value of 20, wall materials were added to the initial solution. These materials comprised MD and a blend of MD and AG (90:10 w/w). Samples without TA were also prepared. In total, four different treatments were prepared as follows:

Powder 1 (P1): anthocyanin + TA + MD + AG.

Powder 2 (P2): anthocyanin + MD + AG.

Powder 3 (P3): anthocyanin + TA + MD.

Powder 4 (P4): anthocyanin + MD.

After preparing the mixture, they were spray‐dried (Maham Sanat, Neyshabur, Iran). The drying parameters were as follows: inlet temperature: 172°C, outlet temperature: 62°C, two‐fluid crossflow nozzles, each with a diameter of 105 cm. Injection flow was 1.2 L/h, and compressor pressure was 1.5 bar. The powders were then kept in dark bottles at −18°C for further analysis.

### 2.5 Total Monomeric Anthocyanin

Total monomeric anthocyanin (TMA) content was determined using the pH differential method described by Lee et al. (2005). The analysis was conducted using a UV/Vis spectrophotometer. A 0.1 mL aliquot of the extract was diluted separately in two buffer solutions: 0.025 M potassium chloride buffer (pH 1.0) and 0.4 M sodium acetate buffer (pH 4.5). After a 15‐min incubation period at room temperature, absorbance was measured at 514 nm (the maximum absorbance wavelength for sour cherry anthocyanins) and at 700 nm (to correct for haze and turbidity). The anthocyanin concentration was expressed as milligrams of cyanidin‐3‐glucoside equivalents per liter (mg CyE/L), calculated using a standard equation.

The absorbance was calculated based on Equation (1):

(1) $$∆A={\left[{\left(A_{514}-A_{700}\right)}_{\mathrm{pH}1}-\left(A_{514}-A_{700}\right)\right]}_{\mathrm{pH}4.5}$$

The total anthocyanin content (TAC), expressed as cyanidin‐3‐glucoside equivalents (mg/kg), was calculated using Equation (2):

(2) $$\text{Total monomeric anthocyanin}\;\left(\mathrm{mg}\frac{\mathrm{CyE}}{L}\right)=\frac{\left(∆A\times \mathrm{MW}\times \mathrm{DF}\times 1000\right)}{\left(\varepsilon \times l\right)}$$

∆*A* = the absorbance, MW = molecular weight of anthocyanin (449.2 g/mol), DF = dilution factor, ε = molar extinction coefficient (26,900 L/mol/cm for cyanidin‐3‐glucoside), l = path length, and the factor 1000 allowed the conversion of grams to milligrams (Lee et al. 2005).

### 2.6 Anthocyanin Retention of Powders (%)

According to the method described by Raharjo et al. (2019), spray‐dried powders were reconstituted in distilled water to achieve a final concentration of 20°Brix. Subsequently, 1 mL of this solution was added to 3 mL of either 0.025 M potassium chloride buffer (pH 1.0) or 0.4 M sodium acetate buffer (pH 4.5) and incubated for 15 min at room temperature. The TAC was then measured using the pH differential method, as described earlier. Anthocyanin concentrations before drying (AC_b_) and after drying (AC_a_) were determined for the calculation of anthocyanin retention.

Equation (3) was used for anthocyanin retention amount:

(3) $$\text{Anthocyanin retention}\;\left(\%\right)=\left({\mathrm{AC}}_{b}\right)/\left({\mathrm{AC}}_{a}\right)\times 100$$

### 2.7 Encapsulation Efficiency (%)

To assess the effectiveness of the encapsulation process, TAC and surface anthocyanin content (SAC) were measured according to the method of Dima et al. (2016). For SAC determination, 0.05 g of powder was added to 10 mL of ethanol in a glass tube and vortexed briefly. The mixture was then centrifuged at 5000 rpm for 3 min. The supernatant was acidified to pH 1 using methanol containing 1% HCl, and the absorbance was recorded at 514 nm using a spectrophotometer. To determine TAC, 0.05 g of powder was mixed with 8 mL of distilled water and placed in an ultrasonic bath (Daihan, Korea) for 20 min. The final volume was adjusted to 10 mL with distilled water, followed by vortexing for 20 s. The solution was filtered through Whatman No. 1 filter paper, and the clear filtrate was acidified with methanol containing 1% HCl and adjusted to pH 1. The absorbance of the solution was then measured at 514 nm.

Then, the encapsulation efficiency (%EE) was calculated using Equation (4):

(4) $$\%\mathrm{EE}=\frac{\left(\mathrm{TAC}-\mathrm{SAC}\right)}{\mathrm{TAC}}\times 100$$

### 2.8 Color

The color of the powders was evaluated following the method of Afshari‐Jouybari and Farahnaky (2011). Briefly, powder samples were spread evenly in Petri dishes and placed inside a wooden box illuminated by a controlled light source (standard light bulb). High‐resolution images of the samples were captured using a mobile phone camera. The images were then analyzed using Adobe Photoshop software to extract color parameters for further evaluation.

Color parameters (L*, a*, b*) were checked, and chroma and hue angle were calculated by the following Equations (5) and (6):

(5) $$\text{chroma}={\left[{\left(a^{*}\right)}^{2}+{\left(b^{*}\right)}^{2}\right]}^{0.5}$$

(6) $$\mathrm{Hue}\;\text{angle}=\tan^{-1}\left(b^{*}/a^{*}\right)$$

L* value describes the intensity of brightness, which changed from 0 (black color) to 100 (white color). Parameter a* describes green (−a*)/red (+a*), and parameter b* describes blue (−b*)/yellow (+b*). The chroma value specifies the color purity or saturation, and hue angle indicates the color of samples (*h* angle = 0° or 360°, 90°, 180°, and 270° signifies red, yellow, green, and blue hues, respectively) (Kuck and Noreña 2016).

### 2.9 Bulk Density and Tapped Density

Bulk density is defined as the ratio of mass to the volume occupied by a sample, measured in a graduated cylinder (g/mL). To determine this parameter, 2 g of each treatment were carefully weighed and transferred into a graduated cylinder with a nominal volume of 10 mL. Tapped density was measured by striking the cylinder 100 times, and the final volume was recorded to calculate the density (Sarabandi et al. 2019).

### 2.10 Flowability (Cohesiveness and Compressibility)

The properties of cohesiveness and compressibility of powders are expressed as the Hausner ratio, Equation (7):

(7) HR=TD/BD

And compressibility of powders is expressed as Carr index and can be measured by Equation (8):

(8) CI=1−1/HR

where TD and BD are tapped and bulk density, respectively (Moshfegh et al. 2025).

### 2.11 Solubility

One gram of dried sample was added to 100 mL of distilled water and centrifuged (3000 rpm for 10 min). The supernatant was separated and dried in an oven at 70°C for 24 h. The difference between the weight of the initial sample and the dried supernatant was considered as powder solubility (Cano‐Chauca et al. 2005).

### 2.12 Moisture Content and Water Activity (*a*_w_)

To measure moisture content, the oven method described by Moshfegh et al. (2025) was used. Also, a water activity meter (Novasina, Switzerland) determined the a_w_.

### 2.13 FTIR Spectroscopy

The ATR‐FTIR spectrophotometer (Tensor II, Bruker) enabled the measurement of the FTIR spectrum of the anthocyanin extract, MD, AG, and spray‐dried powders. The spectrum was recorded with a resolution of 4/cm and an accumulation of 32 scans.

### 2.14 X‐Ray Diffraction (XRD)

An X‐ray diffractometer (D8 ADVANCE, Bruker) was utilized to examine the crystallinity of the spray‐dried powders, extracted anthocyanin, MD, and AG. The analysis was conducted using Cu‐Kα radiation at 40 kV and 40 mA. Diffraction patterns were recorded within a 2*θ* range of 5°–45°, with steps of 0.07° at 1 s per step.

### 2.15 Scanning Electron Microscope (SEM)

To analyze the powder morphology, a small amount of each treated sample was mounted onto tape stubs and coated with a thin layer of gold. The samples were then examined using a scanning electron microscope (TESCAN‐Vega3) that operated at an accelerating voltage of 5 kV. Morphological features were analyzed at magnifications ranging from 1000× to 5000×.

### 2.16 Thermal Analysis

Thermal analysis was conducted using a thermal analyzer (Mettler, China) to obtain thermogravimetric (TGA) and derivative thermogravimetric (DTG) curves. The analysis was carried out under a dynamic nitrogen atmosphere at a flow rate of 50 mL/min, with the temperature ramping from 25°C to 600°C at a rate of 10°C/min.

### 2.17 Storage Stability of the Spray‐Dried Powders

The powder was stored under various conditions to evaluate the effects of light and temperature on stability: (1) room temperature with light exposure, (2) room temperature without light, and (3) refrigeration without light. For light‐exposed conditions, the powders were placed in clear 50 mL Falcon tubes and exposed to fluorescent light (3000 lx) at room temperature (21°C). For dark storage, samples were placed in 50 mL Falcon tubes wrapped in aluminum foil and stored either at room temperature (21°C) or in a refrigerator at 4°C. Anthocyanin retention was measured weekly over a 28‐day period (Moshfegh et al. 2025).

### 2.18 Using Powders in Model Beverages and Investigating the Color Stability of the Model Beverages

To evaluate the stability of the powders in a food system, each spray‐dried powder was used as a coloring agent in a model beverage. The beverage was formulated by dissolving the powder (3.6 g/100 g) along with sugar (10 g/100 g) and citric acid (1 g/100 g) in distilled water. The resulting solutions were transferred into amber bottles and stored in the dark at room temperature (21°C) for 49 days. For control samples, anthocyanin extract, with or without TA, was added to the same sugar, citric acid, and distilled water mixture, maintaining an anthocyanin concentration equivalent to that of the treated samples. The samples were categorized as follows:

Beverage 1: Beverage model P1.

Beverage 2: Beverage model P2.

Beverage 3: Beverage model P3.

Beverage 4: Beverage model P4.

Beverage 5: Beverage model extracted anthocyanin (control).

Beverage 6: Beverage model extracted anthocyanin + TA (control).

To assess the color stability of the beverages, the absorbance of the samples was measured at a wavelength of 514 nm using a spectrophotometer (Bassijeh et al. 2020).

### 2.19 Statistical Analysis

Each measurement was performed in triplicate, while color analysis was conducted in six replicates for all samples. Results were expressed as mean ± standard deviation, with statistical significance set at *p* < 0.05. Data were analyzed using SPSS for Windows, version 26.0 (SPSS Inc.). Mean comparisons were conducted using one‐way analysis of variance (ANOVA), followed by Duncan's multiple range test.

## 3 Results and Discussion

### 3.1 Calculation of Copigmentation Effect

The concentration and type of both copigment and anthocyanin are among the most influential factors affecting the copigmentation process (Azman et al. 2022). Key indicators used to evaluate copigmentation include the bathochromic shift, a shift in the maximum absorption wavelength (Δ*λ*_max_, nm), and the hyperchromic effect, which refers to changes in absorbance at the maximum wavelength (∆*A* at *λ*_max_). Moshfegh et al. (2025) examined the impact of various TA concentrations on copigmentation at a constant anthocyanin level, using anthocyanin: TA molar ratios of 1:0.33, 1:0.25, 1:0.17, and 1:0.08. They identified the 1:0.25 ratio as the most effective, which was therefore adopted in the present study.

**FIGURE 1:**
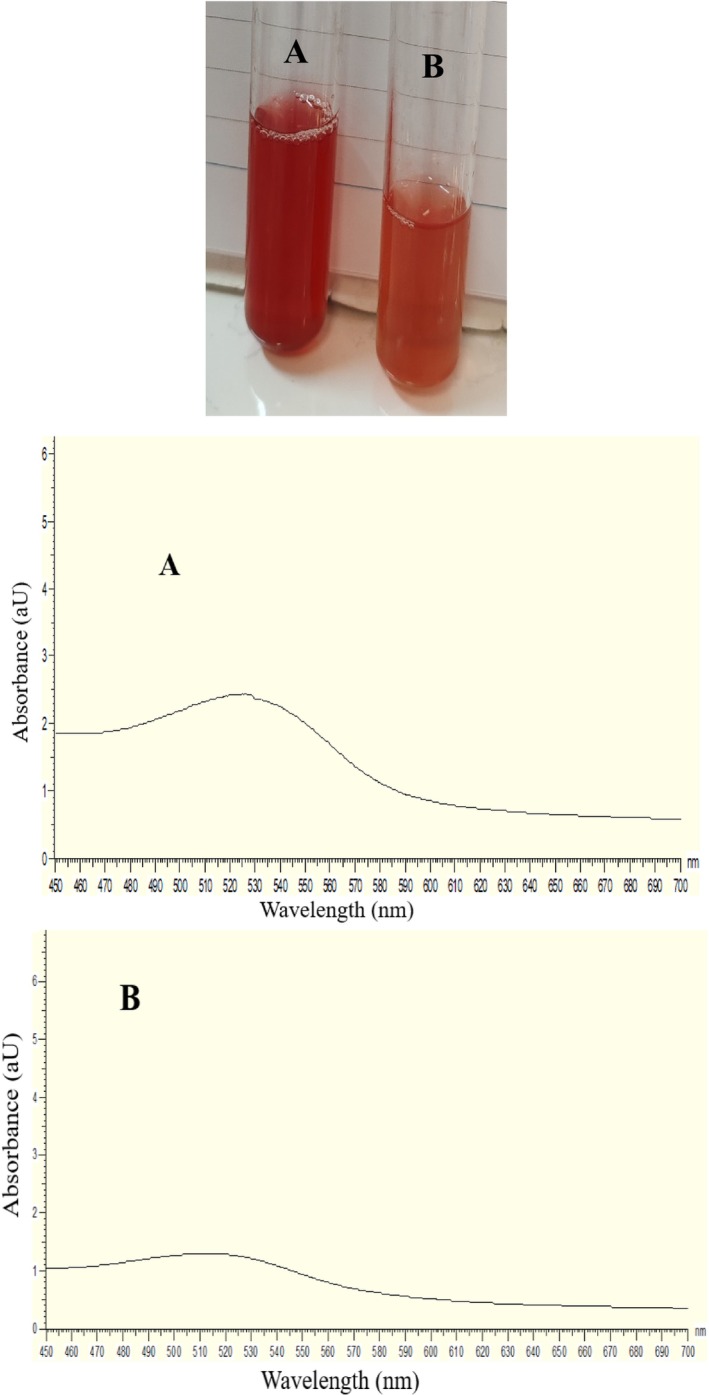
Camera photo and visible spectra of copigment (A) and control (B) solution. (A) Anthocyanin: tannic acid molar ratio of 1:0.25 and (B) anthocyanin: Tannic acid molar ratio of 1:0.

The results of the present study showed that the control sample (without TA) had a maximum absorbance of 1.29 at 511.5 nm (*λ*_max_), whereas the copigmented solution exhibited a maximum absorbance of 2.43 at 524.75 nm (*λ*_max_). This corresponds to a bathochromic shift of 13.25 ± 0.35 nm and a hyperchromic effect with an absorbance increase of 88.68% ± 0.84%. The camera image and visible spectra of both the control and copigmented solutions are shown in Figure 1. Similarly, Li, Bao, et al. (2024) and Li, Feng, et al. (2024) reported that the addition of TA as a copigment to purple sweet potato anthocyanin extract enhanced both absorption peaks and color intensity, confirming the occurrence of copigmentation between TA and anthocyanins.

### 3.2 Total Monomeric Anthocyanin (TMA) and Anthocyanin Retention (%)

In the present study, the TMA content in sour cherries was determined to be 1298.83 ± 9.91 mg CyE/kg. Previous research has reported varying anthocyanin contents across different fruits (Contessa et al. 2013; Fernandes et al. 2015; Mitic et al. 2014). For instance, Contessa et al. (2013) reported the highest anthocyanin concentration in black mulberry at 341.53 mg C3G/100 g fresh weight. Mitic et al. (2014) found that blackberries contained the highest anthocyanin level (1063.53 ± 0.01 mg/kg), while raspberries had the lowest (180.84 ± 0.02 mg/kg). Fernandes et al. (2015) reported anthocyanin contents of 14 mg/L in grape berries and 276.50 mg/kg in cherry extract.

**FIGURE 2:**
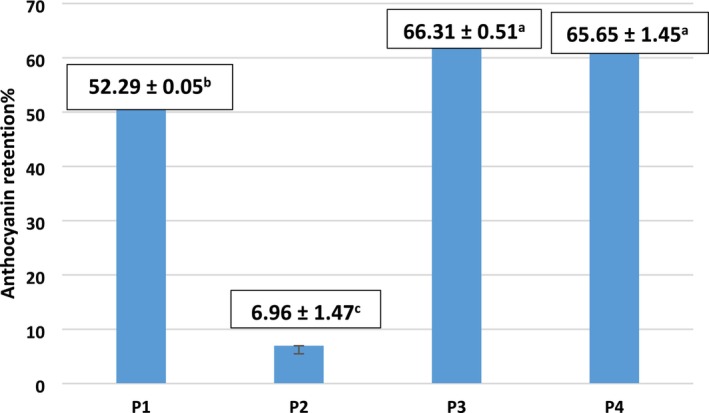
Effects of copigmentation and different carriers on anthocyanin retention (%) of spray‐dried powders. Different letters in each column indicate significant differences between samples (*p* < 0.05). Samples: P1: copigmented anthocyanin + a combination of maltodextrin and Arabic gum (90:10), P2: anthocyanin + a combination of maltodextrin and Arabic gum (90:10), P3: copigmented anthocyanin + maltodextrin, P4: anthocyanin + maltodextrin.

Anthocyanin retention among the powder samples from this study is illustrated in Figure 2. Retention values ranged from 6.96% to 66.31%. The sample produced with MD and AG without copigment (P2) exhibited the lowest retention, while the sample containing MD and TA as copigment (P3) showed the highest retention (*p* < 0.05). Although the difference in retention between MD + TA (P3) and MD alone (P4) was not statistically significant, the formulation containing MD, AG, and TA (P1) retained significantly more anthocyanin than the corresponding sample without copigment (*p* < 0.05). Anthocyanins are known to be heat‐sensitive, and spray drying without protective carriers can result in substantial losses. The use of carriers such as MD during encapsulation promotes the formation of stabilizing interactions with anthocyanins, thereby reducing thermal degradation during spray drying (Moshfegh et al. 2025). According to Moshfegh et al. (2025), copigmentation of sour cherry anthocyanins with TA, followed by encapsulation using either MD or a combination of MD and Persian gum, resulted in anthocyanin retention rates of 53% and 38%, respectively. Similarly, de Araujo Santiago et al. (2016) reported anthocyanin recovery rates of 35% and 59% when pomegranate anthocyanins were encapsulated using MD and AG, respectively.

### 3.3 Encapsulation Efficiency (%)

**FIGURE 3:**
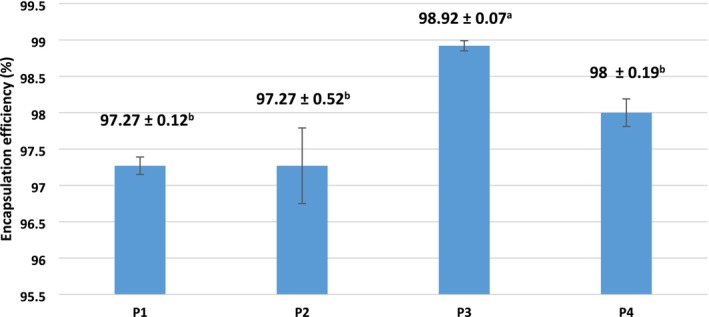
Effects of copigmentation and coating materials on encapsulation efficiency. Different letters in each column indicate significant differences between samples (*p* < 0.05). Samples: P1: copigmented anthocyanin + a combination of maltodextrin and Arabic gum (90:10), P2: anthocyanin + a combination of maltodextrin and Arabic gum (90:10), P3: copigmented anthocyanin + maltodextrin, P4: anthocyanin + maltodextrin.

Encapsulation efficiency reflects the ability of the wall material to entrap and protect the core substance. Higher encapsulation efficiency enhances the protection of anthocyanins from environmental stressors and improves their stability under various storage conditions (Moshfegh et al. 2025). In the present study, all treatments demonstrated high encapsulation efficiencies, ranging from 97.27% to 98.92% (Figure 3). The highest efficiency was observed in sample P3, which incorporated TA as a copigment and MD as the carrier (*p* < 0.05). The other treatments showed no significant differences from one another. Furthermore, powders produced with the combination of MD and AG, whether copigmented or not, did not significantly differ in encapsulation efficiency from noncopigmented samples. These findings suggest that the type of wall material plays a more critical role in encapsulation efficiency than the presence of TA, which did not significantly influence this parameter. Similarly, Nguyen et al. (2022) reported encapsulation efficiencies above 83% when using AG, MD, and their combination for anthocyanin encapsulation, with no statistically significant differences among the treatments (*p* > 0.05).

### 3.4 Color

**FIGURE 4:**
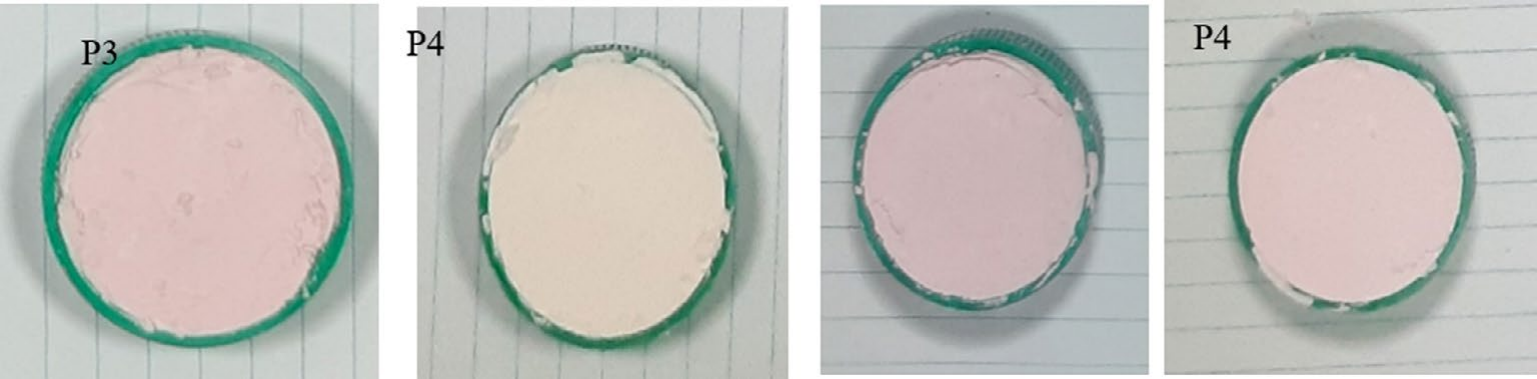
Camera photos of anthocyanin powders. Samples: P1: copigmented anthocyanin + a combination of maltodextrin and Arabic gum (90:10), P2: Anthocyanin + a combination of maltodextrin and Arabic gum (90:10), P3: copigmented anthocyanin + maltodextrin, P4: anthocyanin + maltodextrin.

Color parameters including hue angle (h°), chroma (C*), lightness (L*), and color coordinates (a*, b*), along with camera images of the samples, are presented in Table 1 and Figure 4. The a* and chroma values were significantly higher in treatment P1, indicating enhanced redness and color saturation compared to the other samples (*p* < 0.05). This increase was particularly notable in formulations containing TA and the MD–AG combination, contributing to a visibly redder appearance. In all treatments, a* values remained within the red range. The L* value was highest in P2 (anthocyanin + MD + AG), indicating a lighter or brighter color relative to the other treatments (*p* < 0.05). All samples exhibited positive b* values, signifying yellow tones, with P2 again showing significantly higher yellowness (*p* < 0.05). The hue angles recorded for P1, P2, P3, and P4 were 0.52°, 2.43°, 0.25°, and 0.55°, respectively, with P2 displaying a slightly higher hue angle (*p* < 0.05). Despite this, all samples remained within the red region of the color spectrum. The lower hue angle values in the copigmented samples further underscore the beneficial role of TA in enhancing red color intensity. Jiménez‐Aguilar et al. (2011) also reported hue angles below 10° for blueberry anthocyanin powders encapsulated with mesquite gum, supporting the association between low hue angle and red color dominance. Kalušević et al. (2017) and Moshfegh et al. (2025) found that encapsulation with MD alone resulted in higher a* and chroma values compared to mixtures involving AG or Persian gum. These findings reinforce that the combination of wall materials with copigments significantly enhances the visual and colorimetric quality of anthocyanin‐rich powders.

**TABLE 1:** Color parameters of spray‐dried powders.

Samples	L*	a*	b*	c*	Hue angle
P1 (AN + TA + MD + AG)	81.60 ± 0.50^b^	9.50 ± 0.50^a^	5.10 ± 0.40^b^	10.82 ± 0.43^a^	0.52 ± 0.03^b^
P2 (AN + MD + AG)	84.50 ± 0.50^a^	2.60 ± 0.80^c^	9.50 ± 0.80^a^	9.89 ± 0.86^b^	2.43 ± 1.16^a^
P3 (AN + TA + MD)	76.00 ± 1.60^c^	7.60 ± 0.50^b^	2.00 ± 0.00^d^	7.92 ± 0.49^d^	0.25 ± 0.01^b^
P4 (AN + MD)	81.30 ± 0.70^b^	7.80 ± 0.70^b^	4.30 ± 0.00^c^	9.20 ± 0.63^b^	0.55 ± 0.07^b^

*Note:* Mean ± standard deviation. Different letters in the same column show a significant difference between the samples (*p* < 0.05).

Abbreviations: AG, Arabic gum; AN, anthocyanin; MD, maltodextrin; TA, tannic acid.

### 3.5 Bulk Density and Tapped Density

Bulk density is a crucial parameter in the encapsulation of anthocyanin powders, as it influences their stability, solubility, and accessibility (Nafiunisa et al. 2017; Rosales and Fabi 2022). Moreover, both bulk and tapped densities are economically important due to their implications for transportation and packaging costs. These parameters are directly affected by moisture content, particle shape, and particle size (Darijani et al. 2025; Hoang et al. 2024). In the present study, no significant differences were observed in bulk or tapped density across all treatments (*p* > 0.05) (Table 2). The relatively high bulk densities observed suggest minimal air space between powder particles, which can reduce the potential for oxidation during storage (Carneiro et al. 2013). Shwetha and Preetha (2016), who spray‐dried cumin anthocyanin extracts using MD and AG as wall materials, also reported no clear relationship between the concentration of wall materials and either bulk or tapped density. However, they noted that increasing the concentration of carriers occasionally led to a decrease in bulk density, possibly due to changes in powder structure or porosity.

**TABLE 2:** Characterization of spray‐dried anthocyanin powders.

Samples	Bulk density	Tapped density	Water activity (aw)	Solubility (%)	Moisture content (%)	Hausner ratio	Carr Index
P1 (AN + TA + MD+ AG)	0.33 ± 0.00^a^	0.52 ± 0.00^a^	0.24 ± 0.00^b^	99.99 ± 0.00^a^	2.43 ± 0.35^d^	1.58 ± 0.01^a^	0.37 ± 0.00^a^
P2 (AN + MD + AG)	0.35 ± 0.00^a^	0.52 ± 0.02^a^	0.25 ± 0.00^a^	99.99 ± 0.00^a^	4.05 ± 0.03^c^	1.49 ± 0.06^a^	0.34 ± 0.02^a^
P3 (AN + TA + MD)	0.33 ± 0.00^a^	1.27 ± 0.89^a^	0.22 ± 0.00^c^	99.98 ± 0.00^a^	6.39 ± 0.36^b^	2.04 ± 2.7^a^	0.66 ± 0.24^a^
P4 (AN + MD)	0.34 ± 0.01^a^	0.58 ± 0.02^a^	0.24 ± 0.00^b^	99.99 ± 0.00^a^	7.64 ± 0.11^a^	1.71 ± 0.00^a^	0.40 ± 0.02^a^

*Note:* Mean ± standard deviation. Different letters in the same column indicate a significant difference between the samples (*p* < 0.05).

Abbreviations: AG, Arabic gum; AN, anthocyanin; MD, maltodextrin; TA, tannic acid.

### 3.6 Flowability (Cohesiveness and Compressibility)

The Hausner ratio (HR) is a key indicator for assessing the flowability and packing stability of encapsulated anthocyanin powders. It provides insights into the powders' packing efficiency and agglomeration tendency. Lower HR values correspond to better flowability and dispersibility, which are critical for practical applications in the food and pharmaceutical industries (Bu et al. 2023; Deng et al. 2023). In this study, HR values for samples P1, P2, P3, and P4 were 1.58, 1.49, 2.04, and 1.71, respectively, with no statistically significant differences among treatments (*p* > 0.05) (Table 2). These values suggest that all samples exhibited high cohesiveness and poor flow properties. Generally, an HR value below 1.25 is considered indicative of good flowability (Al‐Hamayda et al. 2023; Darijani et al. 2025). The Carr index is another commonly used parameter for evaluating flow characteristics of powders. Lower Carr index values reflect improved flowability, which is advantageous during handling and processing. In this study, Carr index values for P1, P2, P3, and P4 were 37%, 34%, 66%, and 40%, respectively, with no significant differences observed among the samples (*p* > 0.05). These values further confirm the poor flowability of the encapsulated powders. Moshfegh et al. (2025) also reported similar findings when encapsulating anthocyanins using MD and Persian gum, with comparable values for bulk density, tapped density, HR, and Carr index. In contrast, Girgin et al. (2024) reported significantly better flowability in anthocyanin powders derived from Caucasian blueberries encapsulated with MD, which exhibited a Carr index of 16.7%.

### 3.7 Solubility

Adequate solubility is essential for the effective release and functionality of anthocyanins in various food matrices, as it enhances both their health‐promoting and color‐imparting properties (Nafiunisa et al. 2017; Sakulnarmrat and Konczak 2022). In the present study, the solubility of the encapsulated powders ranged from 99.98% to 99.99%, with no statistically significant differences observed among treatments (*p* > 0.05) (Table 2). These findings indicate that neither the type of wall material nor the presence of TA had a measurable impact on solubility. Consistent with these results, Moshfegh et al. (2025) reported that anthocyanin powders encapsulated with MD, or a combination of MD and Persian gum, with or without TA, showed similarly high solubility and no significant differences between treatments.

### 3.8 Moisture Content and Water Activity

Moisture content is an essential parameter in powder formulations, significantly influencing their storage stability, flowability, and overall quality. Low moisture content enhances the stability of powders by minimizing the risks of caking, clumping, and microbial growth, while high humidity can compromise both physical and microbiological stability (George et al. 2023). In the present study, the moisture content of samples P1, P2, P3, and P4 was determined to be 2.43%, 4.05%, 6.39%, and 7.64%, respectively (Table 2). Samples encapsulated with a combination of MD and AG (P1 and P2) exhibited significantly lower moisture content than those encapsulated with MD alone (P3 and P4) (*p* < 0.05). Furthermore, the copigmented samples (P1 and P3) showed significantly higher moisture content than their noncopigmented counterparts (P2 and P4) (*p* < 0.05). This increase is attributed to the hydrophilic nature of TA, which promotes water retention by forming hydrogen bonds with water molecules (Moshfegh et al. 2025). TA can interact with extracellular proteins, transforming bound water into free water and consequently reducing the overall water‐holding capacity (Wei et al. 2021). Previous studies have also highlighted the impact of carrier type on moisture content. da Silva Carvalho et al. (2016) found that Jusra extract encapsulated with AG alone had higher moisture content than when encapsulated with a mixture of MD and AG. The amphiphilic nature of AG, comprising both protein and polysaccharide components, enables the formation of a protective matrix around moisture and contributes to improved barrier properties. Its film‐forming capacity effectively reduces moisture transfer, enhancing powder stability across different formulations (Jiang et al. 2023).

Water activity is another crucial factor for evaluating powder durability, particularly in terms of microbial and biochemical stability. In this study, water activity values for P1, P2, P3, and P4 were 0.24, 0.25, 0.22, and 0.24, respectively. These values fall below the critical threshold for microbial growth, indicating good shelf stability (Sharifi et al. 2015). The lower water activity observed in copigmented powders further underscores the role of TA in enhancing powder stability by reducing the availability of free water.

### 3.9 FTIR Spectroscopy

**FIGURE 5:**
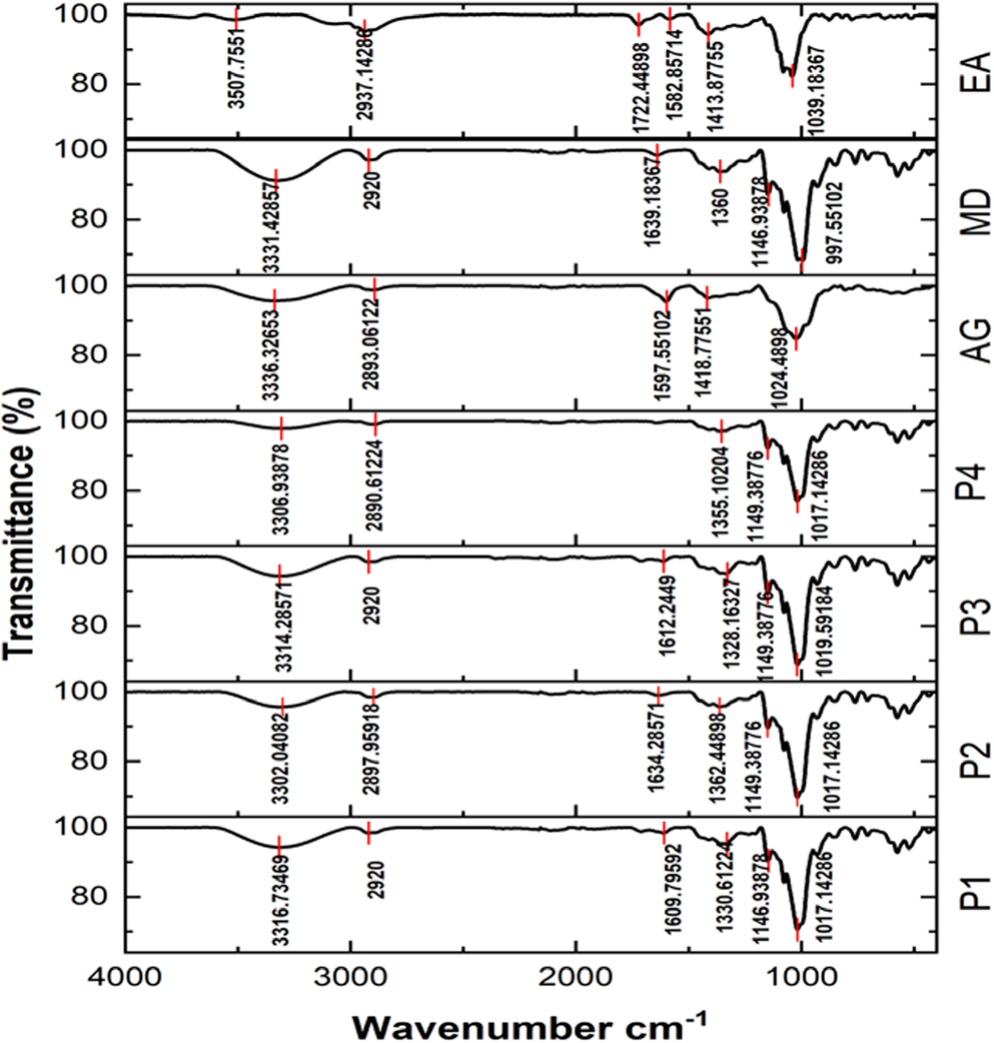
FTIR spectra of (P1): anthocyanin + tannic acid + maltodextrin + Arabic gum, (P2): anthocyanin + maltodextrin + Arabic gum, (P3): anthocyanin + tannic acid + maltodextrin, (P4): anthocyanin + maltodextrin, (MD): maltodextrin, (AG): Arabic gum, (EA): extracted anthocyanin.

FTIR spectrophotometry is a reliable analytical technique for identifying functional groups and assessing structural interactions in treated compounds (Mansour et al. 2020). The FTIR spectra of the anthocyanin extract, wall materials, and encapsulated samples are shown in Figure 5. A broad absorption band observed around 3300 cm^−1^ corresponds to the stretching vibrations of hydroxyl (–OH) and amine (–NH) groups, indicating the presence of phenolic and amino functionalities. A low‐intensity peak around 2925 cm^−1^ is attributed to aliphatic C–H stretching and may also represent carboxyl (–COOH) and carboxylate (–COO^−^) groups, which carry a negative charge (Mansour et al. 2020; Shaddel et al. 2018). The absorption peak near 1150 cm^−1^ is associated with C=C stretching in glucose rings, confirming the presence of sugar moieties. Furthermore, peaks observed at 1039 and 1413 cm^−1^ in the anthocyanin extract are likely due to C–H and C=C stretching in the aromatic ring structure. These are consistent with previous findings by Khadem and Kharaziha (2022), who reported similar peaks at 1080 and 1444 cm^−1^ in red cabbage anthocyanins. A notable peak at 1612 cm^−1^ was observed in treated samples, which may indicate electrostatic interactions between anthocyanins and AG (Mansour et al. 2020). Samples encapsulated with MD and copigmented with TA showed characteristic absorption bands at 3300, 2927, and 1146 cm^−1^, consistent with findings by Moshfegh et al. (2025). For MD, major absorption peaks were observed at 3331 cm^−1^ (O–H stretching), 2920 cm^−1^ (C–H stretching), and 1146 cm^−1^ (C=O stretching), confirming the structural integrity of the wall material. These findings are in agreement with Kang et al. (2019), who reported similar peaks at 3392, 2925, and 1653 cm^−1^ for MD. Overall, the similarity in spectral profiles across all samples suggests that the core anthocyanin compounds were successfully encapsulated within the carrier matrices. Additionally, the presence of characteristic functional group bands for AG and MD in the treated powders implies that the wall materials retained their structural identity following spray drying, thus confirming the effectiveness of the encapsulation process.

### 3.10 X‐Ray Diffraction (XRD)

**FIGURE 6:**
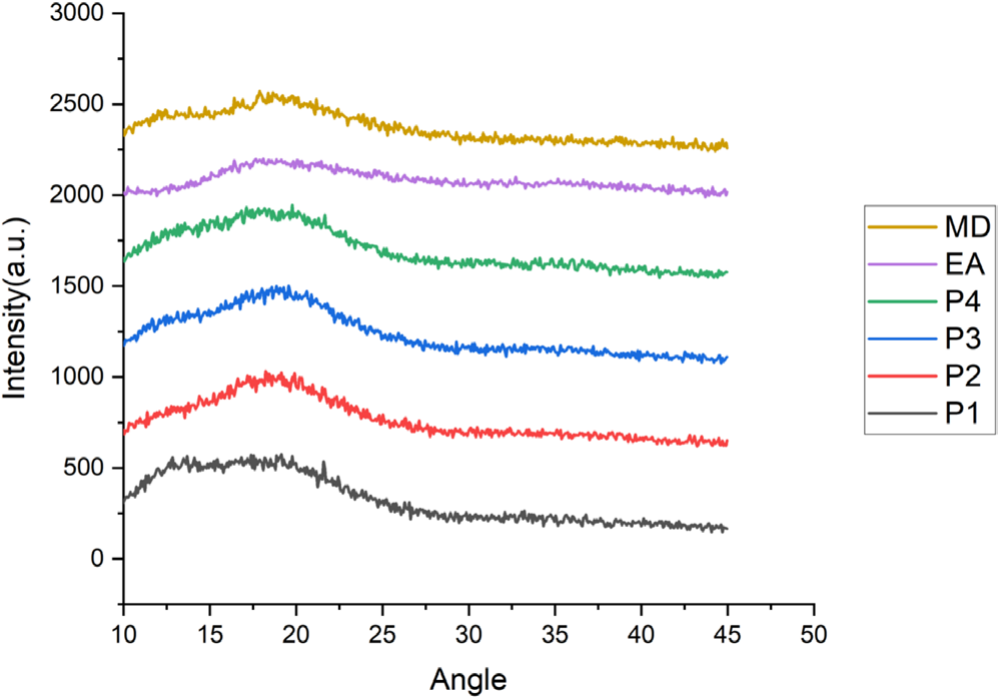
X‐ray diffraction of P1: copigmented anthocyanin + a combination of maltodextrin and Arabic gum (90:10), P2: anthocyanin + a combination of maltodextrin and Arabic gum (90:10), P3: copigmented anthocyanin + maltodextrin, P4: anthocyanin + maltodextrin. EA, extracted anthocyanin; MD, maltodextrin.

XRD analysis plays a critical role in evaluating the crystal lattice arrangement and assessing the degree of crystallinity in powders obtained through encapsulation (Favaro et al. 2018). The XRD patterns of the anthocyanin extract, wall materials, and encapsulated powders are illustrated in Figure 6. All treatments exhibited a broad diffraction peak accompanied by minor noise signals, which are indicative of an amorphous structure. Amorphous materials are known to offer several advantages over their crystalline counterparts, such as enhanced solubility, higher molecular mobility, increased internal energy, and superior thermodynamic stability. In the context of spray drying, the rapid evaporation of the solvent leaves insufficient time for molecular alignment into an ordered crystalline lattice, thereby favoring the formation of amorphous powders. Moreover, both anthocyanins and the employed wall materials, MD and AG, are intrinsically amorphous in nature (Moshfegh et al. 2025). These findings are consistent with previous studies. For example, Tao et al. (2017) encapsulated blueberry anthocyanins using MD, β‐cyclodextrin, and whey protein isolate, and reported broad XRD peaks with multiple low‐intensity signals, confirming the amorphous character of the resulting powders. Similarly, Nthimole et al. (2022) examined freeze‐dried raspberry juice powders using AG, MD, and native starch as wall materials. Their results showed that the powders prepared with AG and MD exhibited predominantly amorphous patterns, while samples encapsulated with native starch displayed characteristic crystalline peaks.

### 3.11 Scanning Electron Microscope

**FIGURE 7:**
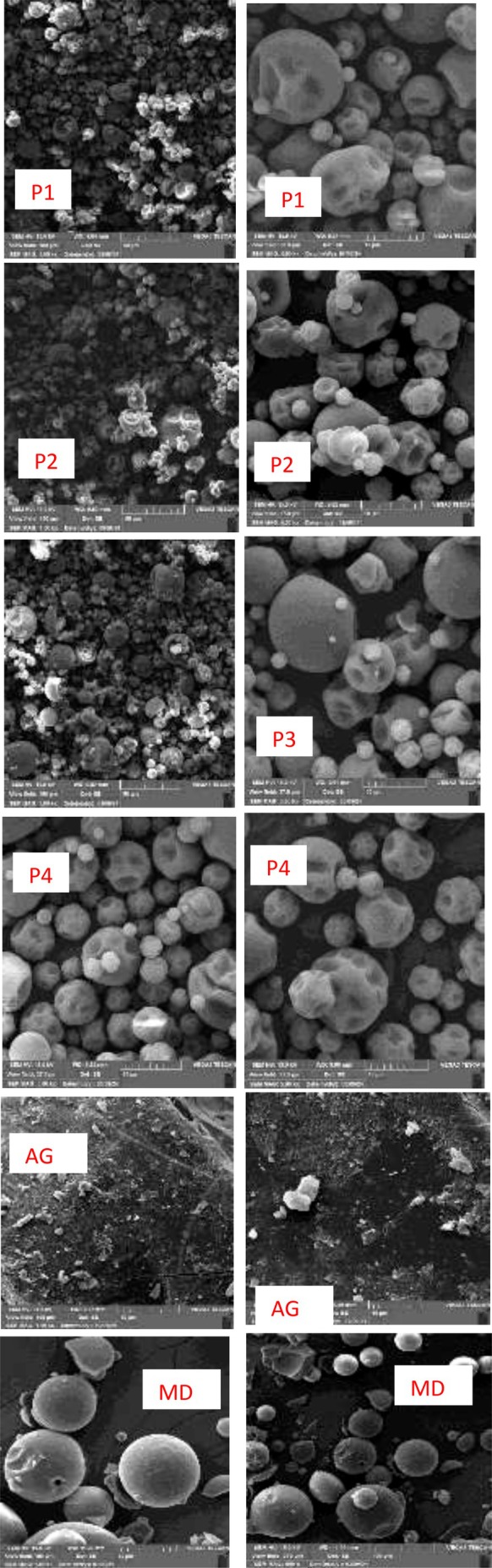
SEM micrographs of samples: P1: copigmented anthocyanin + a combination of maltodextrin and Arabic gum (90:10), P2: anthocyanin + a combination of maltodextrin and Arabic gum (90:10), P3: copigmented anthocyanin + maltodextrin, P4: anthocyanin + maltodextrin. AG, Arabic gum; MD, maltodextrin.

Scanning electron microscope (SEM) images of the encapsulated anthocyanin powders provide valuable insights into the structural characteristics and effectiveness of the encapsulation process (da Silva Carvalho et al. 2016). As shown in Figure 7, the SEM images of the analyzed treatments displayed consistent morphologies, with microcapsule sizes ranging from 10 to 40 μm. Notably, the particle size of treatments P1 and P2 was smaller than that of P3 and P4. In spray drying, the viscosity of the wall material can significantly influence particle size (Kuck and Noreña 2016). In this study, solutions containing both AG and MD exhibited higher viscosity than those containing only MD, which may account for the smaller particle sizes observed in P1 and P2. No significant differences were found between the treatments in terms of concavities and surface wrinkling, indicating that all treatments had similar surface characteristics. SEM images revealed spherical structures with no fissures or cracks, suggesting that the active compounds were successfully encapsulated by the wall materials during the drying process. The observed surface wrinkling is likely a result of the rapid evaporation of atomized droplets during the drying process, particularly at high inlet temperatures (Moshfegh et al. 2025). These findings are consistent with previous studies. For instance, Bulatao et al. (2017) reported similar SEM images showing that anthocyanins extracted from black rice bran were effectively encapsulated using a combination of chitosan and alginate as wall materials via freeze‐drying. The accumulation of wall particles around the core material in their SEM images confirmed the proper encapsulation of the active compounds.

### 3.12 Thermal Analyses

**FIGURE 8:**
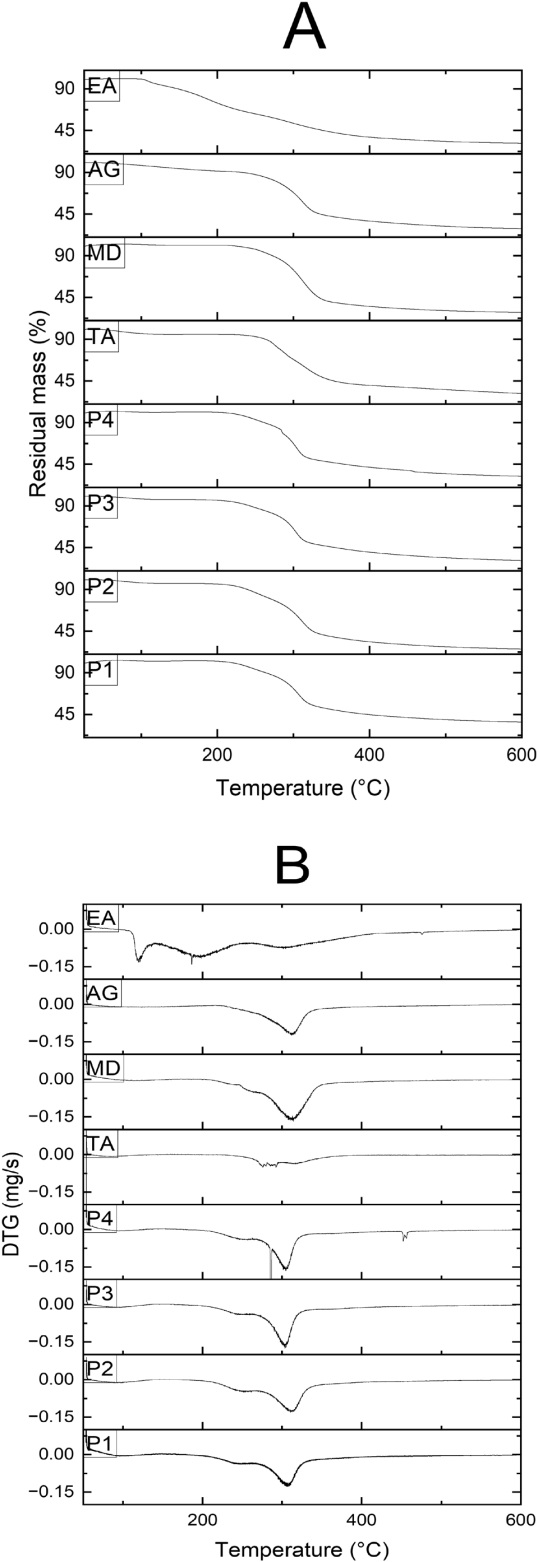
Thermogravimetric curves: (A) TGA and (B) DTG of the treatments: P1: copigmented anthocyanin + a combination of maltodextrin and Arabic gum (90:10), P2: anthocyanin + maltodextrin and Arabic gum (90:10), P3: copigmented anthocyanin + maltodextrin, P4: anthocyanin + maltodextrin. AG, Arabic gum; EA, extracted anthocyanin; MD, maltodextrin; TA, tannic acid.

The thermal behavior of the samples was evaluated using thermogravimetric analysis (TGA) and derivative thermogravimetric (DTG) curves, as presented in Figure 8. TGA assesses the thermal stability of materials by monitoring physical and chemical changes that occur with increasing temperature, particularly through the measurement of weight loss across specific temperature ranges (Moshfegh et al. 2025). In the initial stage of the TGA curves (50°C–110°C), mass loss was primarily attributed to moisture evaporation. Beyond 110°C, thermal decomposition of the sample components commenced (Fritzen‐Freire et al. 2012). In this initial range, no significant mass loss was observed in samples P1 and P4. However, the extracted anthocyanin exhibited approximately 12% mass loss, whereas P2 and P3 showed losses of about 5% and 4%, respectively. Additionally, AG and TA experienced mass losses of 13% and 11%, respectively—values largely attributed to moisture loss. At 350°C, pronounced differences in mass reduction were observed among the samples. MD demonstrated the highest mass loss (65%), followed by the extracted anthocyanin (57%), AG (55%), and TA (55%). In contrast, samples P1, P2, P3, and P4 exhibited mass losses of 44%, 60%, 55%, and 55%, respectively. Among these, P1 had the lowest mass reduction, highlighting the effectiveness of copigmentation and encapsulation with MD and AG in enhancing thermal stability. At 600°C, P1 retained 36% of its initial mass, more than any other treatment, further supporting the role of carrier materials in improving the thermal resistance of extracted anthocyanins during drying.

DTG analysis of the extracted anthocyanin revealed three distinct peaks at 121°C, 196°C–198°C, and 302°C, corresponding to significant thermal degradation events. In contrast, DTG curves for P1, P2, P3, and P4 showed major decomposition peaks at 247°C, 305°C, 303°C, and 304°C, respectively, indicating that the encapsulated treatments provided enhanced thermal stability compared to the unencapsulated anthocyanin extract.

### 3.13 Storage Stability of the Spray‐Dried Powders

In this study, the stability of anthocyanins in the samples was evaluated on days 0, 7, 14, 22, and 28 under three different storage conditions. By day 28, significant differences in anthocyanin retention were observed between P1 and P2, as well as between P3 and P4 (*p* < 0.05), with P1 and P3 showing higher absorbance values than P2 and P4. These results suggest that the presence of TA significantly enhanced anthocyanin retention compared to treatments without copigmentation. However, no significant difference was observed between the carriers used, indicating that MD alone and the MD‐AG combination had similar effects on anthocyanin stability after 28 days. Under light storage conditions, P2 demonstrated more consistent absorbance values throughout the storage period (Table 3). This observation aligns with the findings of Hay et al. (2025), who reported that the addition of AG to MD did not improve the stability of anthocyanins extracted from Antidesma erostre during storage. In contrast, Mahdavi et al. (2016) microencapsulated barberry extract using three different wall materials, MD, MD + AG, and MD + gelatin, via spray drying, and found that all formulations improved shelf life, with the MD + AG combination providing the highest coating efficiency and the lowest anthocyanin degradation rate. Similarly, de Araujo Santiago et al. (2016) encapsulated pomegranate juice extract using AG, modified starch, and MD. Their results showed that a 1:1 combination of AG and modified starch retained 90% and 70% of anthocyanin content after 90 and 120 days of storage at 25°C, respectively. Together, these findings and those of the present study highlight the importance of selecting appropriate wall materials or copigments to enhance anthocyanin stability under various storage conditions.

**TABLE 3:** Anthocyanin retention (%) of spray‐dried powders in days 0, 7, 14, 22, and 28 under 3 different conditions.

Conditions	Room, without light					Room, with light					Refrigerated, without light				
Samples	Day0	Day7	Day14	Day22	Day28	Day0	Day7	Day14	Day22	Day28	Day0	Day7	Day14	Day22	Day28
P1 (AN + TA + MD + AG)	0.28 ± 0.00^bA^	0.21 ± 0.00^aC^	0.18 ± 0.00^bC^	0.22 ± 0.00^aB^	0.24 ± 0.00^aB^	0.28 ± 0.00^bA^	0.21 ± 0.00^aC^	0.18 ± 0.00^bC^	0.22 ± 0.00^aB^	0.24 ± 0.00^aB^	0.28 ± 0.00^bA^	0.23 ± 0.05^aA^	0.17 ± 0.01^bA^	0.22 ± 0.00^aA^	0.22 ± 0.00^bA^
P2 (AN + MD + AG)	0.18 ± 0.02^cA^	0.14 ± 0.00^bA^	0.13 ± 0.00^cA^	0.14 ± 0.00^bA^	0.16 ± 0.02^bA^	0.18 ± 0.02^cA^	0.14 ± 0.00^bA^	0.13 ± 0.00^cA^	0.14 ± 0.00^bA^	0.16 ± 0.02^bA^	0.18 ± 0.02^cA^	0.11 ± 0.01^bB^	0.11 ± 0.00^cB^	0.14 ± 0.00^bB^	0.14 ± 0.00^cB^
P3 (AN + TA + MD)	0.19 ± 0.02^cB^	0.23 ± 0.02^aAB^	0.20 ± 0.00^aB^	0.24 ± 0.00^aA^	0.26 ± 0.01^aA^	0.19 ± 0.020^cB^	0.23 ± 0.02^aAB^	0.20 ± 0.00^aB^	0.24 ± 0.00^aA^	0.26 ± 0.01^aA^	0.19 ± 0.020^cB^	0.20 ± 0.00^abB^	0.22 ± 0.00^aAB^	0.24 ± 0.00^aA^	0.23 ± 0.00^aA^
P4 (AN + MD)	0.35 ± 0.01^aA^	0.14 ± 0.00^bC^	0.09 ± 0.00^dD^	0.12 ± 0.00^bC^	0.17 ± 0.01^bB^	0.35 ± 0.01^aA^	0.14 ± 0.00^bC^	0.09 ± 0.00^dD^	0.12 ± 0.00^bC^	0.17 ± 0.01^bB^	0.35 ± 0.01^aA^	0.11 ± 0.02^bB^	0.07 ± 0.00^dC^	0.12 ± 0.00^bB^	0.13 ± 0.00^cB^

*Note:* Mean ± standard deviation. Different lowercase letters in the same column show a significant difference between the samples (*p* < 0.05). Different uppercase letters in the same row and in each condition show a significant difference between the samples (*p* < 0.05).

Abbreviations: AG, Arabic gum; AN, anthocyanin; MD, maltodextrin; TA, tannic acid.

### 3.14 Investigating Color Stability of the Model Beverages

The results of this study demonstrated that the absorbance of model beverage samples at a wavelength of 514 nm, measured by spectrophotometry at 0, 14, 35, and 49 days, remained nearly constant throughout the 49‐day storage period. To assess potential color changes, the difference in absorbance between days 49 and 0 was calculated. The results revealed no significant differences among the various samples in terms of color change (*p* > 0.05) (Table 4), indicating that both the powder treatments and control samples exhibited comparable color stability in the model beverages. This stability may be attributed to the enhanced stability of anthocyanins under low or acidic pH conditions (pH = 3). Moreover, the findings suggest that under acidic conditions, anthocyanin color stability is inherently favorable, and that neither the type of wall material nor the presence of copigments significantly influenced this parameter. Wang et al. (2024) investigated the use of epigallocatechin gallate, ferulic acid, and gallic acid as copigments to improve anthocyanin stability in blueberry fermented beverages. They found that beverages copigmented with epigallocatechin gallate exhibited the highest absorbance (1.02 a.u.) and the greatest bathochromic shift in maximum absorption wavelength (538 nm) after 90 days of aging, compared to both other copigmented treatments and the control. Similarly, You et al. (2019) evaluated various phenolic acids, including caffeic acid, ferulic acid, *p*‐coumaric acid, gallic acid, *p*‐hydroxybenzoic acid, syringic acid, and TA, as copigments in fresh mulberry juice. Among these, TA resulted in the largest shift in *λ*_max_, from 520 nm to 526 nm. Liu et al. (2019) also studied the use of TA and gallic acid for copigmentation of anthocyanins in bog bilberry syrup wine. They reported that the TAC in the copigmented wines after 6 months of aging was comparable to, or even higher than, that of the three‐month‐aged control wine.

**TABLE 4:** Color stability of the model beverages over time.

Model beverages	Day0	Day14	Day35	Day49	Diff (day 49–0)
Beverage 1 (AN + TA + MD + AG)	0.33 ± 0.00^a^	0.23 ± 0.02^bc^	0.25 ± 0.03^a^	0.19 ± 0.01^bc^	−0.13 ± 0.01^a^
Beverage 2 (AN+ MD+ AG)	0.18 ± 0.00^a^	0.06 ± 0.00^d^	0.05 ± 0.01^f^	0.06 ± 0.08^d^	−0.12 ± 0.00^a^
Beverage 3 (AN + TA + MD)	0.35 ± 0.10^a^	0.29 ± 0.03^a^	0.19 ± 0.00^b^	0.25 ± 0.13^ab^	−0.09 ± 0.13^a^
Beverage 4 (AN + MD)	0.36 ± 0.16^a^	0.17 ± 0.01^c^	0.14 ± 0.01^dcb^	0.14 ± 0.02^cd^	−0.22 ± 0.15^a^
Beverage 5 (AN extract)	0.28 ± 0.00^a^	0.19 ± 0.01^c^	0.17 ± 0.00^eb^	0.18 ± 0.03^bc^	−0.09 ± 0.02^a^
Beverage 6 (AN extract + TA)	0.33 ± 0.16^a^	0.25 ± 0.03^b^	0.16 ± 0.00^c^	0.36 ± 0.01^a^	0.02 ± 0.02^a^

*Note:* Mean ± standard deviation. Different letters in each column indicate significant differences between samples. (*p* < 0.05).

Abbreviations: AG, Arabic gum; AN, anthocyanin; MD, maltodextrin; TA, tannic acid.

## 4 Conclusion

All treatments exhibited high encapsulation efficiency (97.27%–98.92%), with the highest efficiency observed in the copigmented formulation containing TA and MD as the carrier. Despite their favorable solubility, the encapsulated powders showed poor flowability. Notably, the incorporation of TA and AG in the formulations contributed to a reduction in moisture content. XRD analysis confirmed the amorphous structure of all treatments. The combination of MD and AG, particularly when used in conjunction with TA as a copigment, provided the best anthocyanin stability after 28 days of storage. Additionally, the color stability of the model beverages containing anthocyanin powders was comparable to that of control beverages, which is likely due to the acidic pH of the beverage matrix. In conclusion, the findings of this study demonstrate that TA, when used as a copigment alongside MD and AG as wall materials, plays a significant role in preserving anthocyanin content and red color. These materials offer a promising natural alternative to synthetic colorants for use in beverages and other acidic food products.

## Author Contributions

**Shirin Salati:** conceptualization (equal), investigation (equal), methodology (equal), software (equal), writing – original draft (equal). **Niloofar Moshfegh:** investigation (equal), methodology (equal). **Farzaneh Vaseghi‐Baba:** investigation (equal), methodology (equal). **Mehrdad Niakousari:** conceptualization (equal), data curation (equal), methodology (equal), writing – review and editing (equal). **Seyed Mohammad Mazloomi:** data curation (equal), investigation (equal). **Seyed Mohammad Hashem Hosseini:** data curation (equal), investigation (equal). **Azam Abbasi:** conceptualization (equal), funding acquisition (equal), investigation (equal), methodology (equal), supervision (equal), writing – review and editing (equal).

## Conflicts of Interest

The authors declare no conflicts of interest.

## Data Availability

Data will be made available on request.
